# Comparative assessment of external apical root resorption between subjects treated with clear aligners and fixed orthodontic appliances: A systematic review and meta-analysis

**DOI:** 10.34172/joddd.40932

**Published:** 2024-06-24

**Authors:** Swati Singh, Ravindra Kumar Jain, Arthi Balasubramaniam

**Affiliations:** ^1^Department of Orthodontics, Saveetha Dental College and Hospitals, Saveetha Institute of Medical and Technical Sciences, Chennai, Tamil Nadu, India; ^2^Department of Public Health Dentistry, Saveetha Dental College and Hospitals, Saveetha Institute of Medical and Technical Sciences, Chennai, Tamil Nadu, India

**Keywords:** Clear aligners, External apical root resorption, Fixed appliances, Malocclusion, Orthodontic patients

## Abstract

This review aims to collate and analyze the existing evidence on the comparison of external apical root resorption (EARR) in subjects treated with clear aligners (CAs) and fixed appliances (FA). An electronic search was conducted in six databases for articles published in all languages until July 2023. Studies that evaluated EARR in subjects treated with CAs and FAs were included. The RoB 2 tool for RCTs and the ROBINS-I tool for non-randomized studies were used to analyze the risk of bias (ROB). A random effects meta-analysis was performed to assess EARR extent in maxillary and mandibular anterior teeth for subjects treated with CAs and FAs. Ten studies (eight retrospective, one RCT, and one CCT) were included in this review, out of which six studies reported a moderate ROB, one reported a serious ROB, and three reported a low ROB on qualitative analysis. The quantitative analysis of six studies revealed a significant intergroup difference (*P*<0.05) in the mean EARR for maxillary central (SMD=-0.62, *P*<0.00001) and lateral incisors (SMD=-0.47, *P*=0.01) with a moderate heterogeneity (I^2^=56%), as well as the mandibular central incisors (SMD=-0.27, *P*=0.04) with high heterogeneity (I^2^=79%). EARR was lower in subjects treated with CAs than FAs. A moderate quality of the available evidence suggests that EARR was less evident in subjects treated with CAs when compared with FAs.

## Introduction

 External apical root resorption (EARR) is a frequent consequence of orthodontic treatment.^1^ Cemental or surface resorption with remodeling, dentinal resorption with repair, and circumferential apical root resorption with root shortening have been documented as the three types of orthodontically induced inflammatory EARR.^2^ Although orthodontic treatment is an iatrogenic cause of EARR, various other factors, including genetic vulnerability, mechanical causes, and individual biological variance, have been reported.^3,4^

 Subjects undergoing protracted orthodontic treatment involving tooth extraction to gain space are prone to a higher incidence of EARR.^5^ Since most of these changes are irreversible and may impact tooth longevity, clinicians must recognize them during orthodontic treatment.^6^ EARR can be diagnosed using intraoral periapical radiographs (IOPAs), orthopantomograms (OPGs), or cone-beam computed tomography (CBCT).^7^

 Clear aligners (CAs) are transparent removable appliances and are alternatives for fixed orthodontic appliances (FA) to treat malocclusions. They have become very popular recently, particularly among adult patients.^8^ Compared with fixed orthodontic appliances, CAs are more comfortable, aesthetically pleasing, hygienic, and less painful and need fewer and shorter consultations.^8^ CAs are very different from FAs in terms of attachment to the teeth, biomechanics, and treatment duration; hence, we can expect differences in EARR between them.

 Previous systematic reviews have reported on the incidence of root resorption with CA therapy, and a comparison with FAs has been attempted.^9,10^ Both these reviews presented a few limitations and shortcomings; hence, we sought to update the relevant literature and address the limitations. In both reviews, studies assessing EARR individually in CAs and FAs and comparative studies were included, contributing to bias; most studies reported using either OPGs or IOPAs, and a few studies reported CBCTs for examining EARR. Recently, many studies have been published employing CBCTs for evaluating EARR; therefore, it is pertinent to update the existing reviews. The present review aimed to collect and analyze literature specifically on the comparative assessment of EARR following treatment with CA and FA.

 The null hypothesis: There is no difference in EARR in subjects treated with CAs and FA

## Methods

###  Protocol and registration

 The PRISMA 2020 statement’s reporting standards for systematic reviews and meta-analyses were followed in the reporting of this study. This systematic review was submitted to the International Prospective Register of Systematic Reviews - PROSPERO database and given a registration number - CRD42023448412.

###  Search strategy

 An electronic search of the literature published in the below-mentioned databases was carried out to identify all papers related to the research question: Google Scholar, PubMed, Scopus, Cochrane, and Cochrane Embase. Open Grey and GreyNet International were searched for grey literature. Key words were modified for each database (Table 1). The search was conducted for articles published until July 2023 in all languages. Rayyan’s duplicate removal tool was used.^11^

**Table 1:** Search strategy and results for each database

**Database**	**Search Strategy**	**Results**
PubMed	("orthodontal"[All Fields] OR "orthodontic"[All Fields] OR "orthodontical"[All Fields] OR "orthodontically"[All Fields] OR "orthodontics"[MeSH Terms] OR "orthodontics"[All Fields] OR (("orthodontal"[All Fields] OR "orthodontic"[All Fields] OR "orthodontical"[All Fields] OR "orthodontically"[All Fields] OR "orthodontics"[MeSH Terms] OR "orthodontics"[All Fields]) AND ("patient s"[All Fields] OR "patients"[MeSH Terms] OR "patients"[All Fields] OR "patient"[All Fields] OR "patients s"[All Fields])) OR ("tooth movement techniques"[MeSH Terms] OR ("tooth"[All Fields] AND "movement"[All Fields] AND "techniques"[All Fields]) OR "tooth movement techniques"[All Fields] OR ("tooth"[All Fields] AND "movement"[All Fields]) OR "tooth movement"[All Fields]) OR ("malocclusal"[All Fields] OR "malocclusion"[MeSH Terms] OR "malocclusion"[All Fields] OR "malocclusions"[All Fields] OR "malocclusive"[All Fields])) AND ((("clear"[All Fields] OR "cleared"[All Fields] OR "clearing"[All Fields] OR "clearings"[All Fields] OR "clears"[All Fields]) AND ("align"[All Fields] OR "alignability"[All Fields] OR "alignable"[All Fields] OR "aligned"[All Fields] OR "alignement"[All Fields] OR "aligner"[All Fields] OR "aligners"[All Fields] OR "aligning"[All Fields] OR "alignment"[All Fields] OR "alignments"[All Fields] OR "aligns"[All Fields])) OR (("clear"[All Fields] OR "cleared"[All Fields] OR "clearing"[All Fields] OR "clearings"[All Fields] OR "clears"[All Fields]) AND ("align"[All Fields] OR "alignability"[All Fields] OR "alignable"[All Fields] OR "aligned"[All Fields] OR "alignement"[All Fields] OR "aligner"[All Fields] OR "aligners"[All Fields] OR "aligning"[All Fields] OR "alignment"[All Fields] OR "alignments"[All Fields] OR "aligns"[All Fields])) OR ("align"[All Fields] OR "alignability"[All Fields] OR "alignable"[All Fields] OR "aligned"[All Fields] OR "alignement"[All Fields] OR "aligner"[All Fields] OR "aligners"[All Fields] OR "aligning"[All Fields] OR "alignment"[All Fields] OR "alignments"[All Fields] OR "aligns"[All Fields]) OR ("orthodontic appliances, removable"[MeSH Terms] OR ("orthodontic"[All Fields] AND "appliances"[All Fields] AND "removable"[All Fields]) OR "removable orthodontic appliances"[All Fields] OR "invisalign"[All Fields]) OR "orthocaps"[All Fields] OR "clearcorrect"[All Fields]) AND ("orthodontic appliances, fixed"[MeSH Terms] OR ("orthodontic"[All Fields] AND "appliances"[All Fields] AND "fixed"[All Fields]) OR "fixed orthodontic appliances"[All Fields] OR ("fixed"[All Fields] AND "appliance"[All Fields]) OR "fixed appliance"[All Fields] OR ("orthodontic appliances, fixed"[MeSH Terms] OR ("orthodontic"[All Fields] AND "appliances"[All Fields] AND "fixed"[All Fields]) OR "fixed orthodontic appliances"[All Fields] OR ("fixed"[All Fields] AND "orthodontic"[All Fields] AND "appliance"[All Fields]) OR "fixed orthodontic appliance"[All Fields])) AND ((("external"[All Fields] OR "externally"[All Fields] OR "externals"[All Fields]) AND ("apical"[All Fields] OR "apically"[All Fields] OR "apicals"[All Fields] OR "apices"[All Fields]) AND ("root resorption"[MeSH Terms] OR ("root"[All Fields] AND "resorption"[All Fields]) OR "root resorption"[All Fields])) OR (("apical"[All Fields] OR "apically"[All Fields] OR "apicals"[All Fields] OR "apices"[All Fields]) AND ("root resorption"[MeSH Terms] OR ("root"[All Fields] AND "resorption"[All Fields]) OR "root resorption"[All Fields])) OR ("root resorption"[MeSH Terms] OR ("root"[All Fields] AND "resorption"[All Fields]) OR "root resorption"[All Fields]))	62
Scopus	orthodontic patients OR orthodontics OR malocclusion OR tooth movement AND clear aligner OR clear aligners OR invisalign OR aligner OR clearcorrect OR orthocaps AND fixed appliance OR fixed orthodontic appliance AND external apical root resorption OR apical root resorption OR root resorption	63
Cochrane	(orthodontic patients):ti,ab,kw OR (tooth movement):ti,ab,kw OR (malocclusion):ti,ab,kw OR (orthodontics):ti,ab,kw AND (clear aligner):ti,ab,kw (clear aligners):ti,ab,kw OR (aligners):ti,ab,kw OR (clear aligner):ti,ab,kw OR (clearcorrect):ti,ab,kw AND (orthocaps):ti,ab,kw (Word variations have been searched) (fixed appliance):ti,ab,kw OR (fixed orthodontic treatment):ti,ab,kw AND (metal brackets):ti,ab,kw (Word variations have been searched) external apical root resorption):ti,ab,kw OR (apical root resorption):ti,ab,kw OR (root resorption):ti,ab,kw (Word variations have been searched)	21
Cochrane Embase	(orthodontic patients):ti,ab,kw OR (tooth movement):ti,ab,kw OR (malocclusion):ti,ab,kw OR (orthodontics):ti,ab,kw AND (clear aligner):ti,ab,kw (clear aligners):ti,ab,kw OR (aligners):ti,ab,kw OR (clear aligner):ti,ab,kw OR (clearcorrect):ti,ab,kw AND (orthocaps):ti,ab,kw (Word variations have been searched) (fixed appliance):ti,ab,kw OR (fixed orthodontic treatment):ti,ab,kw AND (metal brackets):ti,ab,kw (Word variations have been searched) external apical root resorption):ti,ab,kw OR (apical root resorption):ti,ab,kw OR (root resorption):ti,ab,kw (Word variations have been searched)	4
Google Scholar	orthodontic patients OR orthodontics OR malocclusion OR tooth movement AND clear aligners OR aligners OR invisalign OR clear aligner AND fixed orthodontics OR fixed orthodontic appliance OR fixed mechanotherapy AND external apical root resorption OR apical root resorption OR root resorption	107
Web Of Science	Orthodontic patients AND clear aligners AND fixed appliance AND external apical root resorption	3

###  Eligibility criteria

 Randomized controlled trials, prospective studies, and retrospective studies were included. Review articles, letters to the editor, and case reports/series studies were excluded. Studies involving patients treated with both CAs and FAs in separate groups were included. Both before and after treatment, IOPA, OPG, or CBCT had to be available to measure EARR. In all the included studies, only subjects with dental root maturity were assessed. EARR had been reported in the included studies as a percentage or mm change in root length after treatment:

- Population: Subjects undergoing orthodontic treatment
- Intervention: Clear aligners
- Control: Fixed appliances
- Outcome: External apical root resorption

###  Screening and selection of studies

**Figure 1:**
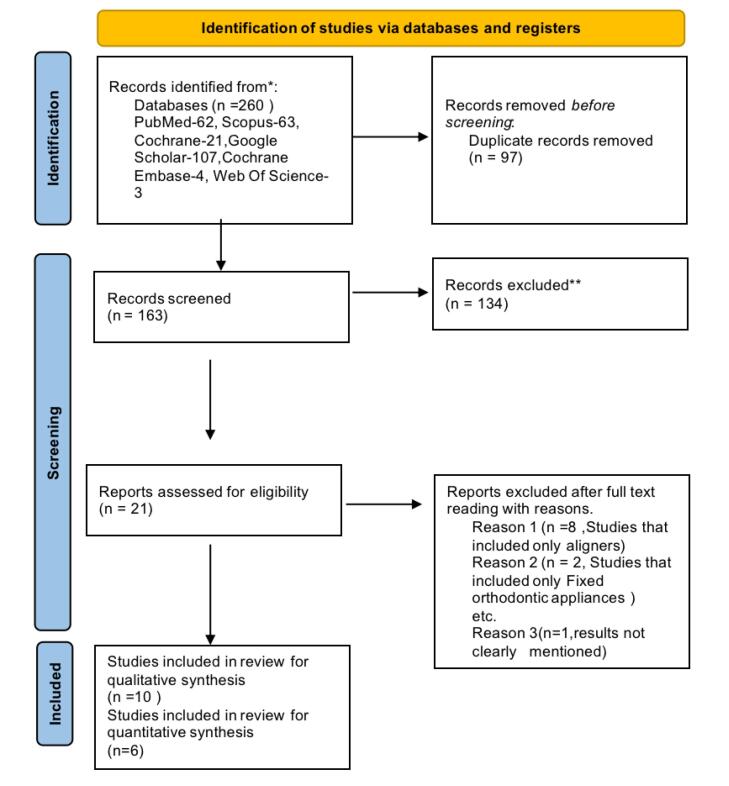
PRISMA flow diagram for study selection

 All studies that met the selection criteria were included in the review. The PRISMA flow chart shows the procedure for choosing the studies for the review (Figure 1). The selection of the studies, tabulation, and the RoB assessment were performed by both authors (SS and RKJ). Any differences were discussed with the third author (AB) and resolved. The characteristics table included the following general data from the included studies: First author’s name, the year and the journal of publication, teeth evaluated, sample size, outcomes assessed, and the evaluation method.

###  Outcome measures

 EARR was measured as a millimeter change in apical root length assessed on IOPA/OPG/CBCT or measured as a volumetric change on CBCT.

###  Qualitative assessment of the included studies

**Figure 2:**
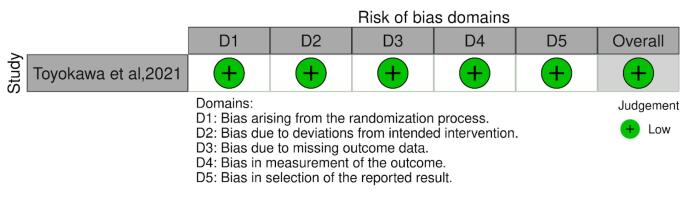
Cochrane RoB 2 for RCTs

**Figure 3:**
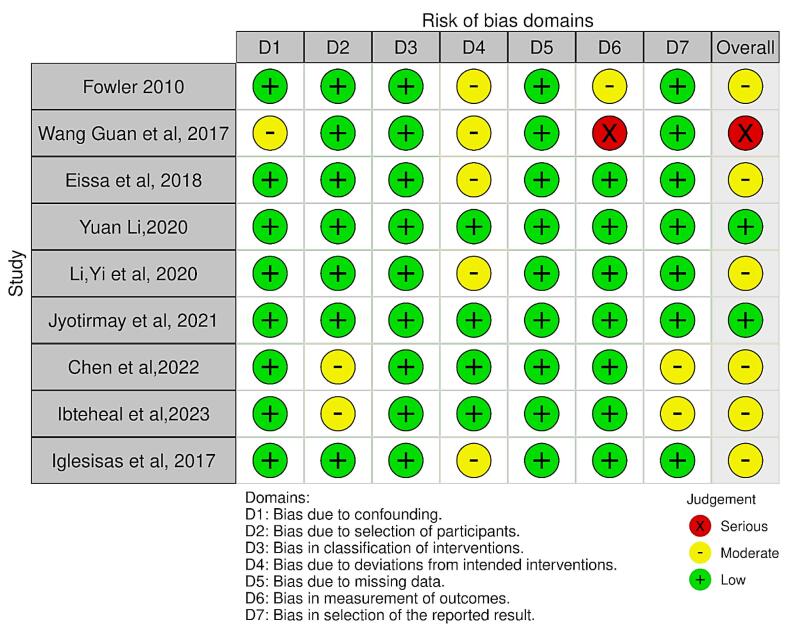
ROBINS-I RoB for retrospective studies

 The Cochrane RoB 2 tool was used for RCTs, while the ROBINS-I tool was utilized for qualitative evaluation of non-randomized trials. The biases included by the ROBINS-I tool are confounding, bias in participant selection, bias in the classification of interventions, bias due to deviations from intended interventions, bias introduced by missing data, bias in outcome measurement, bias in result reporting, and overall bias. When assessing the bias in the RCT, bias resulting from the randomization process, bias due to deviations from the intended interventions (effect of assignment to intervention), bias due to missing outcome data, bias in the measurement of the outcome, and bias in the selection of the reported result were all considered. Each signaling question was reported as “partially yes,” “yes,” “no,” “partially no,” or “not indicated.” The ROB analysis of the retrospective studies is summarized in Figures 2 and 3. Both authors (SS and RKJ) worked independently and reviewed all the included studies for their qualitative assessment. Any conflict was resolved by mutual discussion.

###  Quantitative assessment of the included studies

 Using the Cochrane Review Manager software (Revman Web), a meta-analysis of the mean and standard deviation of the primary outcome was carried out. A random effects model (DerSimonian-Laird random effects pooling approach) was used to compute the overall effects. A subgroup meta-analysis with a pooled mean difference was conducted. The EARR was quantified as a mean mm or %. Some of the articles included various measuring tools for EARR. A funnel plot was used to determine publication bias.

###  Statistical analysis 

 The outcome measure evaluated was EARR, measured as a reduction in root length in mm or percentage. Outcome data of root length reduction in mm was pooled from the included studies and analyzed using weighted mean difference with a 95% confidence interval. Heterogeneity was assessed using I-squared (I^2^) tests. Sensitivity analysis was performed by deleting each study individually to detect the quality and consistency of the results. A random-effects model of meta-analysis was used for quantitative analysis. Forest plot analysis was used to determine the influence of each subgroup on heterogeneity. A funnel plot analysis was performed to assess publication bias. Revman Web software was used for the analysis mentioned above.

## Results

###  Study Selection 

 Authors SS and RKJ individually performed the screening and selected the studies according to the selection criteria. Any disagreements were resolved by consultation with the third author AB. The electronic search identified 260 articles. After removing the duplicates (n = 97) using the Rayyan software, 163 studies were assessed for title and abstract screening, of which 142 articles were excluded. Of the 21 studies, after excluding 11 studies for various reasons, 10 studies were included in the qualitative analysis, and out of them, six were subjected to quantitative analysis (as depicted in Figure 1).

###  Study characteristics 

 The detailed characteristics of the included studies are presented in Table 2, providing all details about the authors, study design, number of participants, treatment duration, type of malocclusion, and the outcomes assessed (root resorption in maxillary and mandibular anterior teeth). One was a randomized control trial,^12^ one was controlled clinical trial (CCT),^13^ and eight were retrospective clinical trials.^14-21^ Three studies assessed EARR in both maxillary and mandibular anterior teeth.^16,17,21^

**Table 2:** Characteristics table of the included studies

**Author, year of publication**	**Study design**	**Treatment duration**	**Evaluated teeth**	**Study groups/sample size with Intervention**	**Type of malocclusion**	**Outcomes Assessed **
None et al, 2021^16^	Retrospective cohort	CA: 22.23 ± 7.34 months FA: 22.45 ± 6.54 months	MX and MN anterior teeth	CA- 55(Inline aligners); FA- 55 (3M, USA)	Subjects with dental crowding, proclination, and spacing (both extraction and non-extraction cases)	EARR assessment on CBCT as root length reduction
Toyokawa-Sperandio et al, 2021^12^	Parallel group RCT	6 months after the start of orthodontic therapy	MX and MN incisors	CA- 20 SmartTrack, Invisalign TM; Align Technology, San Jose, CA, USA; FA- 20 slot 0.022” × 0.030”, 3M Unitek, Monrovia, CA, USA	Angle’s Class I malocclusion with mild crowding and non-extraction therapy	EARR on IOPA
Yi et al, 2018^14^	Retrospective cohort	CA: 21.54 ± 5.55 months FA: 23.31 ± 6.25 months	MX and MN anterior teeth	CA- 35 Invisalign, Align Technology, California, USA FA- 35 Victory Series; 3 M Unitek, California, USA CA- (Invisalign, Align Technology, California, USA)Series; 30 FA- (Victory Series; 3 M Unitek, California, USA). (males-21, females-49)	Extraction and non-extraction cases	Prevalence and severity of EARR on CBCTs
Eissa et al, 2018^15^	Pilot study	CA:15.14 ± 1.94 months DQ-FA: 15.75 ± 1.74 months 3M FA: 16.27 ± 2.74 months	MX incisors	CA- 11 SmartTrack®(San Jose, California, USA); FA- 11 in preadjusted edgewise bracket(3M Unitek, California, USA). Damon-Q self-ligating brackets-11 (Ormco Corporation 1717)	Angles Class I malocclusion (mild to moderate crowding) (non-extraction	EARR using CBCT.
Li et al 2018^17^	Retrospective study	CA: 22.08 ± 4.51months FA: 20.83 ± 5.29 months	MX incisors	CA- Clear thermoplastic aligners FA- PEA with 0.022 slots	Non-extraction (mild to moderate crowding)	EARR using OPG.
Chen et al, 2022^18^	Pilot study	CA: 30.94 ± 4.32 months FA: 28.30 ± 5.08 months DQ-FA: 27.67 ± 4.72 months	MX CI	CA- 18 (Invisalign, Align Technology, Calif) FA- 20, 0.022-in slot Victory Series; 3M Unitek, Calif; Damon Q with a 0.022-in slot-21 (DQ; Ormco, Orange, Calif)	Angle Class II Division II malocclusion, None extraction cases (moderate crowding)	EARR using CBCT
Almagrami et al 2023^19^	Retrospective comparative study	CA: 25.85 ± 8.0 months FA: 29.67 ± 7.71 months	MX incisors	CA- 20(Align Technology, California, USA) FA- 20(Victory Series; 3 M Unitek®, California, USA)	Mild to moderate crowding, non-extraction treatment	EARR using CBCT
Wang et al 2017^20^	Retrospective study	CA: 1.5 ± 0.3 years FA: 1.7 ± 0.4 years	MX and MN incisors	CA- 28(Invisalign-USA) FA- 28(3M Victory Series, USA)	Angles Class I malocclusion with mild to moderate crowding, non-extraction	EARR using CBCT
Iglesias-Linares, 2017^13^	Case-Control genetic association study	FA: 30.73 ± 12.37 months CA: 29.56 ± 11.64 months	MX incisors	CA- Invisalign, Align Technology, San Jose, California FA- straight wire technique, CEOSA DM, Madrid	Extraction and Non-extraction cases, Angles Class I, II, III malocclusion	Frequency of EARR using OPG
Fowler et al, 2010^21^	Retrospective cohort	FA: 19.69 months CA: 20.36 months	MX and MN anterior teeth	CA- 45, Invisalign FA- 45, 0.022-inch slot appliance, MBT prescription	Non-extraction cases, Angles Class I malocclusion	EARR using panoramic radiographs

FA: fixed appliance, CA: clear aligner, EARR: external apical root resorption, MX: maxillary, MN: mandibular, CI: central incisor, LI: lateral incisor, C: canine.

 Four studies assessed EARR in maxillary incisors,^11,12,13,14^ whereas one reported EARR only in maxillary central incisors.^17^ Two studies assessed maxillary and mandibular incisors.^12,20^ None of the included studies reported EARR assessment for premolars and molars. Six studies reported EARR assessment based on CBCT,^15-20^ one on IOPAs,^10^ and three used OPGs.^13,14,21^ Nine of the included studies reported on EARR in mm or percentage loss of the root length,^12-17,19-21^ and one study reported volume change.^18^ Two studies included extraction and non-extraction cases,^13,17^ and the rest included subjects with mild to moderate crowding treated without extractions.^12,14-16,19-21^

 Invisalign was used in seven studies,^12,13,15,17,18,20,21^ and other aligners were reported in the rest.^14,16,19^ In all studies, the subjects were treated with FAs using MBT 0.22 metal brackets in the control group. EARR in CAs ranged from 0.14 ± 0.53 to 2.66 ± 1.46 mm, whereas in FAs, it was in the range of 0.55 ± 0.54 to 2.63 ± 1.46 mm. The results of the studies are presented in Table 3.

**Table 3:** Results of the included studies

**Author and Year of Study**	**Results (*****P***** value)**	**Inference**
None J et al, 2021^16^	The mean value of EARR in FA was 1.51 ± 1.34 mm, and in CA was 1.12 ± 1.34 mm (*P* < 0.001)	CAs showed less EARR than FAs.
Toyokawa-Sperandio et al, 2021^12^	Intergroup comparisons of EARR (CA: −0.52 ± 0.57; FA: −0.86 ± 0.60 ) revealed a significant difference for MX CI (*P* = 0.037)	CAs and FAs resulted in a similar degree of EARR.
Yi et al, 2020^14^	The prevalence of EARR in CA (56.30%) was less than FA(82.11%) (p < 0.001). The severity of EARR in CA (0.13 ± 0.47 mm) was less than in FA (1.12 ± 1.34 mm) (*P* = 0.001).	The patients treated with CAs had a lower prevalence and severity of EARR than FA.
Eissa et al, 2018^15^	Cases treated with FA(1.04 ± 0.67) showed significantly higher EARR than those treated with SmartTrack® aligners. (0.44 ± 0.35) (*P* < 0.05)	Subjects treated with CAs showed less EARR.
Li et al, 2018^17^	Compared to the FA group (6.97 ± 3.67%), the mean value of EARR in the CA group was much lower at (5.13 ± 2.81%) which was statistically significant (*P* < 0.05).	Subjects treated with CAs had reduced EARR
Chen et al, 2022^18^	Root length and volume loss were less in CA (23.68 ± 4.82 mm^3^) followed by FA (28.24 ± 6.44 mm^3^) which was statistically significant (*P* < 0.05)	CA-treated subjects showed less incidence of fenestration and root resorption
Almagrami et al, 2023^19^	EARR in the CA group was significantly less (0.31 ± 0.42) than those in the FA group (0.68 ± 0.97) (*P* < 0.000)	CAs and FAs appear to cause a significant alveolar bone thickness reduction. Increased EARR in the maxillary incisor region with FAs was noted.
Wang et al, 2017^20^	EARR occurred in 47.3% of teeth in the CA group and 68.8% in the FA group with a significant intergroup difference (*P* < 0.05).	CAs resulted in less root resorption than FAs.
Iglesias-Linares, 2017^13^	The final associations between clinical and genetic factors and predisposition to EARR showed no statistically significant differences (*P* > 0.05) about EARR or the type of orthodontic appliance used (OR: 1.662; 95% CI: 0.945–2.924; *P* = 0.078)	EARR in subjects treated with CAs was similar to FAs.
Fowler et al, 2010^21^	—	Less EARR in subjects treated with CAs

EARR: External apical root resorption; CA: Clear aligners; FA: Fixed Orthodontic Appliances; OR: Odds Ratio, CI: Confidence interval.

###  Risk of bias analysis

 The RCT by Toyokawa-Sperandio et al^12^ had a low risk of bias (ROB) as assessed by the RoB-2 tool (Figure 2). ROB assessment for retrospective studies was performed using the ROBINS-I tool, which revealed a moderate ROB for six studies.^13-15,18,19,21^ low risk for two studies,^16,17^ and serious^20^ concerns for one study (Figure 3).

###  Result of the qualitative analysis

 Eight of the ten studies reported less EARR with CAs than FAs.^14-21^ The remaining two studies reported similar EARR in patients treated with both CAs and FAs.^12,13^ The mean root resorption for the permanent maxillary incisors was in the range from 0.26 to 2.66 mm and 0.23 to 1.31 mm for permanent maxillary lateral incisors, and 0.20 to 0.73 mm for mandibular central incisors, which were clinically significant.

###  Result of quantitative analysis

**Figure 4:**
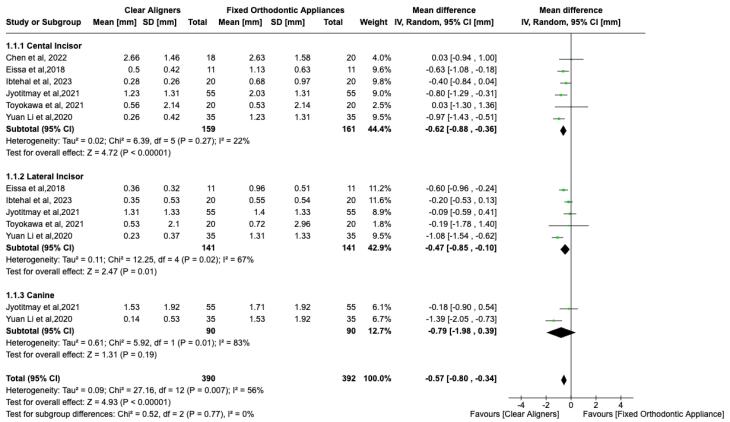
Forest plot of EARR in the maxillary anterior teeth

 The meta-analysis of the six included studies^12,15-19^ revealed a significant mean difference between CAs and FAs for maxillary central incisors (SMD = -0.62, 95% CI: -0.88, -0.36, P < 0.01) (Figure 4). The overall heterogeneity across the studies was moderate (I^2^ = 56%); thus, a random-effects model was used for quantitative assessment. Figure 4 shows a significant standard mean difference between CAs and FAs for lateral incisors (SMD = -0.47, 95% CI: -0.85, -0.10, *P* < 0.01), favoring CAs. However, no significant difference was noted for canines (SMD = -0.79, 95% CI: -1.98, -0.39, *P* > 0.01). With a moderate heterogeneity (I^2^ = 56%), there was a significant difference in the overall effect between CAs and FAs, favoring Cas (Figure 4).

**Figure 5:**
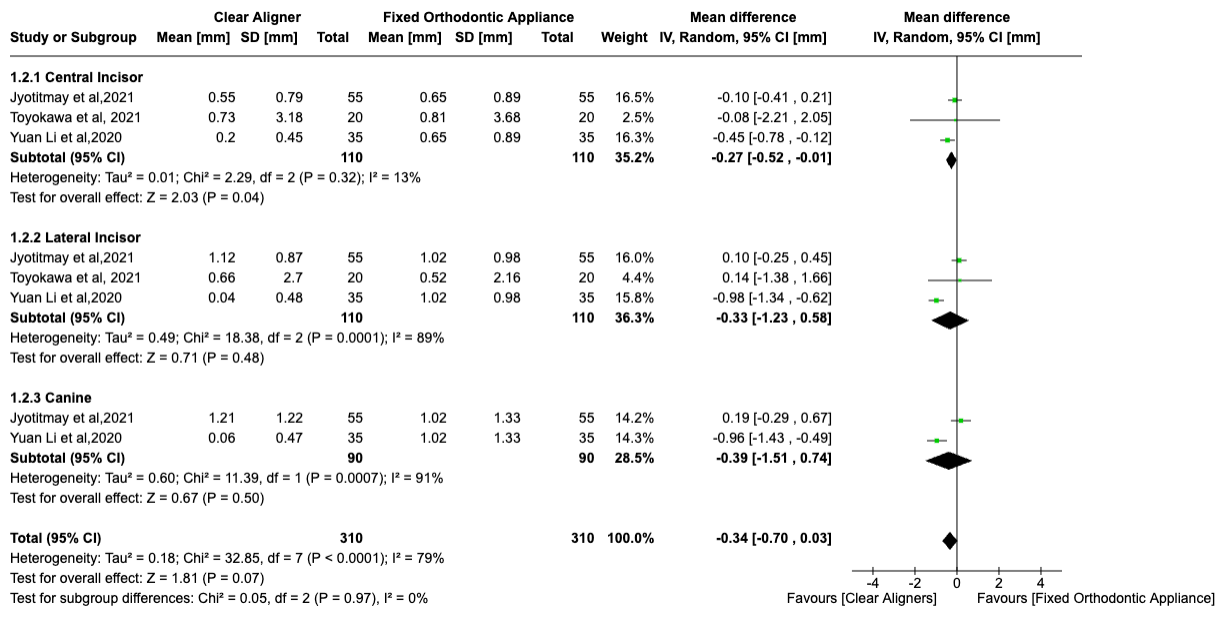
Forest plot of EARR in the mandibular anterior teeth

**Figure 6:**
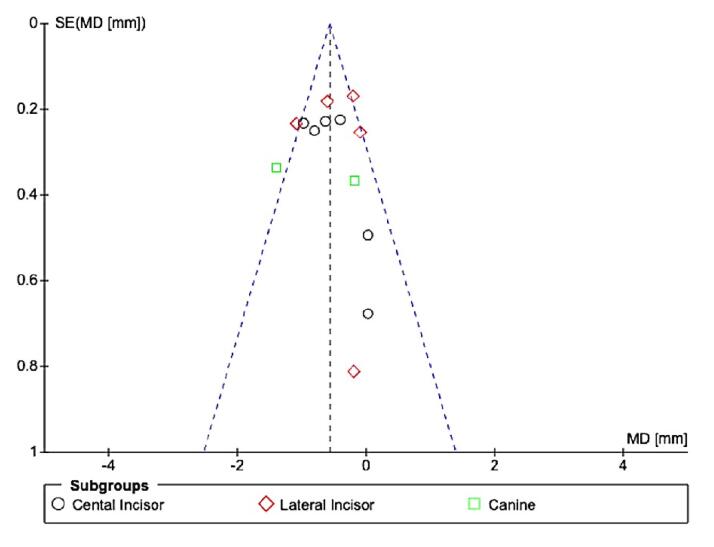
Funnel plot for publication bias of studies reporting on EARR of maxillary anterior teeth

**Figure 7:**
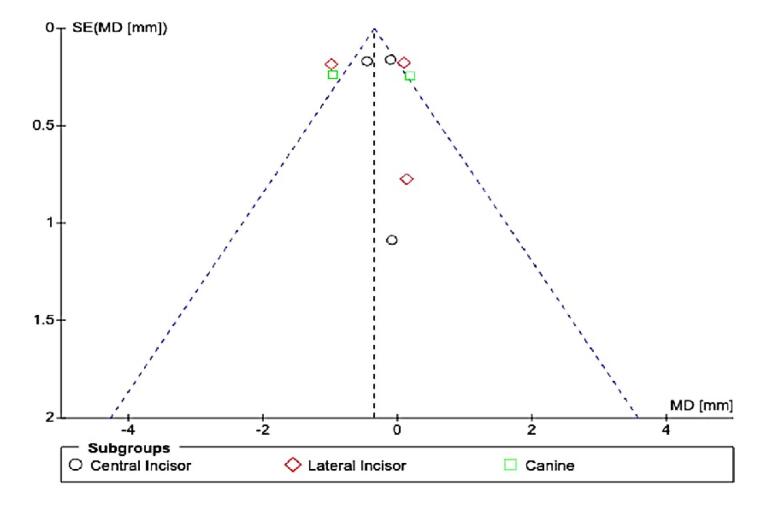
Funnel plot for publication bias in studies that report EARR of mandibular incisors

 For mandibular anterior teeth, there (Figure 5) was no significant mean difference between CAs and FAs for EARR with high heterogeneity (I^2^ = 79%). However, there was a significant difference in the central incisor (SMD = -0.27, 95% CI: -0.52, -0.01, P < 0.01), with no difference for lateral incisors (SMD = -0.33, 95% CI: -1.23 to 0.158) and canines (SMD = -0.39, 95% CI: -0.70 to 0.03). The overall effect neither favored CAs nor FAs. Possible publication bias was noted, especially for maxillary canines, and mandibular lateral incisors, and canine assessments, since the standard error was high in one study (Figure 6).^12^ Possible publication bias was noted, especially for mandibular lateral and central incisor assessment, since the sample size was small (Figure 7).^15^

## Discussion

 EARR is a well-documented consequence of orthodontic treatment involving both fixed and removable appliances. This systematic review was carried out to gather, compile, and analyze data on the extent and severity of EARR in patients treated with CAs and compare it with patients treated with FAs from human clinical trials published as retrospective studies and randomized and non-randomized trials. This review involved only studies with CAs as an intervention and FAs as a comparison or control group. A total of ten studies reported on EARR of various teeth individually with IOPA, CBCT, and OPG in millimeters, percentages, or volume. Both qualitative and quantitative analyses revealed a lower overall incidence of EARR in subjects treated with CAs than in those treated with FAs. On analyzing the individual teeth for EARR, it was noted that maxillary central and lateral incisors had less EARR in subjects treated with CAs than in subjects treated with FAs. Concerning mandibular teeth, central incisors had less incidence of EARR in subjects treated with CAs than in subjects treated with FAs.

 Different mechanical aspects between CAs and FAs contribute to changes in EARR. Aligners, if well tolerated by patients, result in predictable tooth movement, leading to less EARR when compared with FAs.^22^ With aligners, forces created are light and intermittent as they are removed during food consumption to maintain oral hygiene. With interrupted forces, less EARR was observed during orthodontic treatment according to the study by Sawicka et al,^23^ and the risk of EARR increases by recurrent tooth movement brought on by jiggling forces. Non-extraction treatment strategy, a shorter course of treatment, and less root movement during orthodontic therapy may contribute to non-significant EARR with either CA or FA.^9,10^ FAs are better at torque control/ buccolingual inclination and root angulation than CAs. Hence, we can infer that since torque control, buccolingual movements, and root movements are less effective with CAs, the incidence of EARR is also less with CAs.^24^ Also, preplanning and staging various tooth movements is done in CAs; hence, the possibility of multiple tooth movements at a single point in time, subjecting the teeth to unpredictable forces, is very remote. CAs rely on patient compliance; hence, whenever patients are not using them, cementum repair takes place, which is not seen with FAs.^25^ In one of the investigations, it was reported that refinements during CA treatment reduce the risk of EARR. Because CAs are worn intermittently and retainers are prescribed before refinements, repair following root resorption is achievable with CAs. This may also be one of the causes of the decreased prevalence of EARR in CAs.^14^

 Most of the retrospective studies included in this review had a moderate to low ROB, while the RCT had a low ROB. Deviation from intended interventions contributed to bias in some of the included retrospective studies. Previous published systematic reviews on the comparison of root resorption between CA and FA reported some limitations; hence, this review was undertaken. Previously published reviews have not included studies with both CA and FA groups. A review by Fang et al.^9,10^ included three studies employing CBCT as a tool for EARR assessment, whereas six studies in this review reported using CBCT.CBCT is more reliable for measuring EARR than IOPAs or OPGs. In the present study, CBCT data from five studies were subjected to a subgroup meta-analysis of individual teeth. Two studies included in this review had EARR data of extraction cases with higher crowding and treatment duration, reducing bias.^17,19^ The systematic review by Gandhi et al^9^ included studies that investigated EARR in patients who underwent FA therapy without extractions using FAs or CAs by either CBCT or 2D radiographic examination, unlike the present review.^10^ Except for two studies in their review, patients treated with either CAs or FAs were evaluated separately for EARR. In the present ystematic review, all the included studies had subjects treated with CAs and FAs, hence avoiding pooling data from different studies in different setups and on different populations.

 In the present review, the quantitative assessment of EARR between CAs and FAs showed a significant mean difference for maxillary central incisor and lateral incisor with less incidence of EARR in CAs, which are not consistent with the findings of a review by Gandhi et al.^9^ Although there was no significant difference between FA and CA for EARR of teeth #22, #12, and #23 in their review (*P* > 0.05), the subjects treated with FA had significantly more root resorption than the subjects treated with CA for upper right lateral incisor teeth. In the review by Fang et al,^10^ individual incisor teeth showed significantly less EARR with CAs compared to FA for all maxillary incisors, which is consistent with the present study. As treatment duration is a factor influencing EARR, it was noted in the present review that in all included studies, the treatment duration was roughly similar for subjects treated with both CAs and FAs.^9,10^ The treatment duration or the assessment period for CAs in the included studies was as short as six months^11^ and as long as 30.94 ± 4.32 months,^19^ and for FA, it was as short as six months^11^ and as long as 29.67 ± 7.7 months. Therefore, intergroup differences in treatment duration will not influence the severity of EARR. Root volume was measured in only one of the included studies, and it was shown that participants who received CAs showed much less root volume reduction than those who received FAs. Additionally, the CA group experienced a higher increase in labial bone thickness at the apical level.^19^

## Limitations

 Only one RCT was included, and most included studies were retrospective with a moderate ROB. The results of this research must be employed carefully because separate investigations have used various diagnostic modalities for the same examined measurements, which contributes to heterogeneity. The included articles involved a combination of different aligners and both conventional edgewise and pre-adjusted edgewise and self-ligating appliances, which might lead to differences in root resorption.

## Conclusion

 The following conclusions can be drawn from the present review considering its limitations:

1. Moderate-quality evidence suggests that the extent and severity of EARR were lower in subjects treated with CAs than in subjects treated with FAs.
2. On quantitative assessment, maxillary central and lateral incisors and mandibular central incisors exhibited significantly less EARR in subjects treated with CAs.

## Competing Interests

 None declared by the authors. This paper has not been published elsewhere nor submitted elsewhere for publication.

## Ethical Approval

 Approval for this study (SRB/SDC/ORTHO-2103/21/078) was provided by the Institutional Scientific Review Board of Saveetha Dental College, Chennai. This systematic review was submitted to the International Prospective Register of Systematic Reviews - PROSPERO database and given a registration number - CRD42023448412.

## Funding

 Public, private, or non-profit funding organizations did not provide any grants for this research, and it is a self-funded research.
